# A helical LC3-interacting region mediates the interaction between the retroviral restriction factor Trim5α and mammalian autophagy-related ATG8 proteins

**DOI:** 10.1074/jbc.RA118.004202

**Published:** 2018-10-03

**Authors:** Jeremy R. Keown, Moyra M. Black, Aaron Ferron, Melvyn Yap, Michael J. Barnett, F. Grant Pearce, Jonathan P. Stoye, David C. Goldstone

**Affiliations:** From the ‡ School of Biological Sciences, University of Auckland, Auckland 1010, New Zealand,; the § Francis Crick Institute, London NW1 1ST, United Kingdom,; the ¶ School of Biological Sciences, University of Canterbury, Christchurch 8041, New Zealand, and; the ‖ Maurice Wilkins Centre for Molecular Biodiscovery, Auckland 1010, New Zealand

**Keywords:** autophagy, retrovirus, human immunodeficiency virus (HIV), protein-protein interaction, protein complex, antiviral protein, LC3B, LIR, retroviral restriction factor, Trim5alpha

## Abstract

The retroviral restriction factor tripartite motif–containing 5α (Trim5α) acts during the early postentry stages of the retroviral life cycle to block infection by a broad range of retroviruses, disrupting reverse transcription and integration. The mechanism of this restriction is poorly understood, but it has recently been suggested to involve recruitment of components of the autophagy machinery, including members of the mammalian autophagy-related 8 (ATG8) family involved in targeting proteins to the autophagosome. To better understand the molecular details of this interaction, here we utilized analytical ultracentrifugation to characterize the binding of six ATG8 isoforms and determined the crystal structure of the Trim5α Bbox coiled-coil region in complex with one member of the mammalian ATG8 proteins, autophagy-related protein LC3 B (LC3B). We found that Trim5α binds all mammalian ATG8s and that, unlike the typical LC3-interacting region (LIR) that binds to mammalian ATG8s through a β-strand motif comprising approximately six residues, LC3B binds to Trim5α via the α-helical coiled-coil region. The orientation of the structure demonstrated that LC3B could be accommodated within a Trim5α assembly that can bind the retroviral capsid. However, mutation of the binding interface does not affect retroviral restriction. Comparison of the typical linear β-strand LIR with our atypical helical LIR reveals a conservation of the presentation of residues that are required for the interaction with LC3B. This observation expands the range of LC3B-binding proteins to include helical binding motifs and demonstrates a link between Trim5α and components of the autophagosome.

## Introduction

The ATG8-like proteins are essential for expansion of the phogophore membrane and mediate targeting and assembly of protein complexes to the autophagosome (1). The six mammalian ATG8 (mATG8)^3^ isoforms (LC3A, LC3B, LC3C, GABARAP, GABARAPL1, and GABARAPL2) are orthologues of the *Saccharomyces cerevisiae* ATG8 protein and share a conserved sequence and structure. Structurally, the mATG8s consist of a β-grasp fold that is shared with ubiquitin and ubiquitin-like proteins (2). In addition, mATG8s have two further helices at the N terminus that complete the fold. A conjugation pathway similar to the ubiquitin conjugation system results in the proteins being C-terminally lipidated with phosphatidylethanolamine, providing an anchor to the autophagosomal membrane. As key components of selective autophagy, they can act to target specific proteins to the autophagosome together with proteins and organelles targeted for destruction within the autophagosome.

The mATG8s bind target proteins through a conserved LC3-interacting region (LIR) (3). The mATG8s, and their interaction with the LIR motif, has been well-characterized by structural biology (2, 4–6). The typical LIR motif is formed by a large hydrophobic residue and a small hydrophobic residue separated by two intervening amino acids that are not arginine, glycine, proline, or lysine. It is often accompanied by an acidic residue at the N-terminal end of the motif giving the following prosite (7) motif annotation [DEST][WFY]-{RGKP}{RGKP}-[ILV]. Upon binding, the LIR motif forms an extended β-strand that extends the central β-sheet packing parallel to strand β2. Similarly a GABARAP-interacting motif has recently been described where a small hydrophobic residue is located immediately following the large hydrophobic residue (8). The large and small hydrophobic residues of the LIR are accommodated within two hydrophobic pockets located on the surface of the ATG8 protein (2) The first pocket, often termed the W pocket, accommodates the large hydrophobic residue, and the second pocket, often termed the L pocket, accommodates the small hydrophobic residue.

Recently, several studies have described a link between members of the TRIM (tripartite motif–containing) protein family and autophagy (9–13). Members of the TRIM protein family are characterized by a conserved N-terminal domain architecture consisting of a RING domain, which confers E3 ubiquitin ligase activity; one or two Bbox domains; and a coiled-coil region (14). The C-terminal domain of TRIM proteins is varied with a PRY/SPRY domain being the most common (15). The coiled-coil region assembles as an elongated anti-parallel dimer placing the RING and Bbox domains from each monomer at opposite ends of the coiled-coil, separating them by ∼160 Å (16–18). Family members act in many cellular pathways, with approximately one-third implicated in innate immunity (19–22). One of the most studied family members is the antiretroviral postentry restriction factor Trim5α.

Trim5α acts during early stages of the retroviral life cycle to prevent retroviral infection, disrupting reverse transcription and integration of the virus. The restriction of a particular virus requires recognition of the intact lattice of capsid protein that forms the inner shell of the retrovirus (23, 24). Recognition of the incoming virus is mediated by the C-terminal domain PRY/SPRY domain. A set of variable loops in the PRY/SPRY domain dictates the subset of viruses that the Trim5α of different species is able to restrict (25).

Trim5α blocks infection during at least two stages of the retroviral life cycle. The first block, prior to reverse transcription, is contingent on the ubiquitin-proteasome system and results in premature disassembly of the capsid core and release of viral proteins and RNA (26). Inhibition of the proteasome or disruption of the Trim5α RING domain prevents Trim5α from blocking reverse transcription but does not rescue infection, indicating the presence of a second block to infection (27, 28).

In 2014 Mandell *et al.* (9) used a siRNA screen to show that a large number of TRIM proteins alter the number of LC3B puncta in cells, suggesting a role in regulating autophagy. In these experiments Trim5α was proposed to act as a selective autophagy receptor, targeting a restricted virus to the autophagosome for degradation. Furthermore, using co-immunoprecipitation they demonstrated potential interactions with components of the autophagic machinery including ULK1, beclin1, sequestosome1/p62, and members of the mATG8 family.

To investigate the interaction between Trim5α and components of the autophagy machinery, we have undertaken *in vitro* experiments, using purified proteins, to examine the interaction between members of the mammalian ATG8 family and the coiled-coil region of Trim5α. We have demonstrated a direct interaction and determined the strength and stoichiometry of this interaction. Furthermore, we have crystallized the complex between Trim5α and LC3B. Our structure demonstrates that a cryptic LIR is located in the Trim5α coiled-coil α-helix and that an LIR need not be present as a β-strand or disordered region of the protein. Although retroviral infection assays demonstrate that the interaction is not required for restriction of HIV-1, this structure demonstrates an expanded range of binding sites for LC3B and members of the mammalian ATG8 proteins and provides a structural link between Trim5α and components of the autophagosome.

## Results

### Trim5α binds directly to the six mammalian ATG8 isoforms

The interaction between mATG8 family members and Trim5α was identified by co-immunoprecipitation studies (9). Further peptide array experiments then mapped binding to regions of the Trim5α coiled-coil domain. This suggests an atypical interaction, because ATG8 proteins usually bind an LIR motif found in a β-strand or unstructured loop. To address this discrepancy, we sought to reproduce the interaction with purified components.

**Figure 1.**
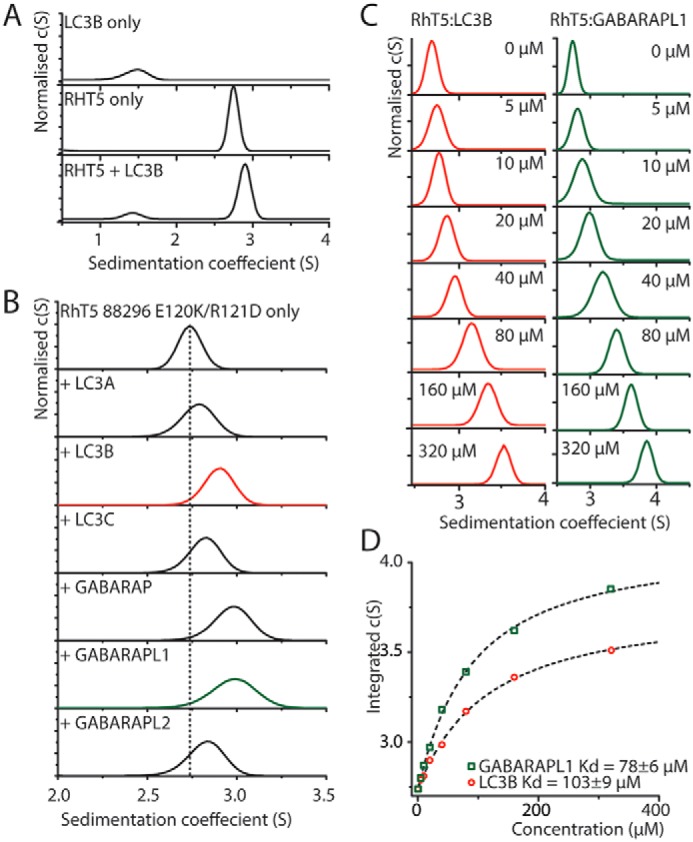
**SV–AUC analysis shows that purified Trim5α binds to six isoforms of mATG8s.** *A*, *c*(*s*) analysis of SV–AUC of 20 μm LC3B, RhT5 88–296 EK/RD, or an equimolar mixture. *B*, *c*(*s*) analysis of 20 μm of RhT5 88–296 EK/RD and equimolar concentration of the six mATG8 isoforms. *C*, *c*(*s*) analysis of 20 μm RhT5 88–296 EK/RD and increasing concentrations (0–320 μm) of either LC3B or GABARAPL1. *D*, peak centroid position derived from integration of the *c*(*s*) function from *C versus* LC3B or GABARAPL1 concentration. A one-site binding model has been used to determine the equilibrium dissociation constant (*dashed lines*).

To do this, we utilized a construct of Trim5α from Rhesus macaque encompassing the Bbox coiled-coil regions with an E120K/R121D mutation (RhT5 88–296 EK/RD) that blocks the higher-order assembly of the protein (16, 29), and LC3B expressed in *Escherichia coli*. Our initial small-scale pulldown experiments failed to recapitulate the interaction. Therefore, we employed sedimentation velocity analytical ultracentrifugation (SV–AUC) to examine the interaction between the Trim5α coiled-coil and LC3B where the two components were closer to equilibrium conditions. Analysis was undertaken using the continuous distribution of sedimentation coefficients function, *c*(*s*), for each component alone and an equimolar mixture of RhT5 88–296 EK/RD and LC3B (Fig. 1*A*). The *c*(*s*) distribution for RhT5 88–296 EK/RD and LC3B showed symmetric peaks with sedimentation coefficients of 2.74 S (*S*_20,w_ 2.86 S) and 1.56 S (*S*_20,W_ 1.7 S), respectively, consistent with RhT5 88–296 EK/RD being a dimer and LC3B being a monomer. Analysis of the mixture showed a slow-moving peak at 1.56 S corresponding to free LC3B and a fast-moving peak at 2.90 S. This peak is at a greater *S* value than that seen for RhT5 88–296 EK/RD alone and represents the unresolved co-sedimentation of both the RhT5 88–296 EK/RD and the 88–296 EK/RD+LC3B complex components. Because no peak was observed for free RhT5 88–296 EK/RD, this suggests that the interaction is under fast exchange in solution relative to the time of sedimentation (30).

To determine whether the interaction with Trim5α is common to all mATG8s, we undertook further sedimentation velocity experiments employing RhT5 88–296 EK/RD and LC3A, LC3C, GABARAP, GABARAPL1, and GABARAPL2 and analyzed the effect on the position of the fast-moving peak. In each case the addition of a mATG8 resulted in an increase in the apparent sedimentation coefficient in the integrated *c*(*s*) of the RhT5 88–296 EK/RD peak. Based upon the magnitude of the change in the peak position, that likely corresponds to the strength of the interaction, the rank order of affinity was GABARAPL1 followed by GABARAP, LC3B, GABARAPL2, and LC3C, with LC3A being the weakest (Fig. 1*B*).

To measure the affinity of the interaction, two representative mATG8 proteins, LC3B and GABARAPL1, were chosen and titrated (0–320 μm) against a 20 μm RhT5 88–296 EK/RD and analyzed binding by SV–AUC. In both the LC3B and GABARAPL1 titrations, a concentration-dependent shift was observed for the fast-moving species with the S value of the peak increasing as the concentration of either protein was increased. To determine the affinity of the interaction, the *S* value of the faster moving peak in the *c*(*s*) distribution was integrated and plotted against the ATG8 protein concentration. The curve was then fit to a single site-binding model. The equilibrium dissociation constant (*K_D_*) determined in this manner was 103 ± 9 and 78 ± 6 μm for LC3B and GABARAPL1, respectively (Fig. 1, *C* and *D*).

### LC3B binds directly to the Trim5α coiled-coil via helical LIR motif

To identify the site of interaction of mATG8s within the Trim5α coiled-coil, we undertook crystallization experiments employing RhT5 88–296 EK/RD and all the mATG8s. Crystals of a complex between RhT5 88–296 EK/RD and LC3B were obtained and harvested for X-ray diffraction analysis. The crystals diffracted anisotropically to a resolution of 4.11–2.74 Å and belong to the space group P22_1_2_1_. The structure was determined by molecular replacement using the structure of the Bbox coiled-coil region of Trim5α (PDB code 4TN3) and LC3B (PDB code 3WAO) as search models. The structure was refined to a final *R*/*R*_free_ of 25.9%/27.4%, respectively. Two copies of RhT5 88–296 EK/RD and two copies of LC3B are present in the asymmetric unit (full data collection and model refinement statistics are presented in Table 1). The final model comprises residues 95–288 of RhT5 and residues 88–296 and 4–117 of LC3B.

**Table 1:** **Data collection and refinement statistics** The statistics for the highest resolution shell are shown in parentheses.

	RhT5 88–296 E120K/R121D: LC3B 2–119
**Data collection statistics**	
Diffraction source	MX2 Beamline, Australian Synchrotron
Space group	P 2 21 21
Unit cell dimensions	
*a*, *b*, *c* (Å)	72.01, 115.32, 174.16
α, β, γ (°)	90, 90, 90
Resolution range (Å)	40.73–2.74 (2.97–2.74)
Ellipsoidal resolution (Å) (direction)*^a^*^,^*^b^*	2.723 (a*)
	2.768 (b*)
	4.111 (c*)
Total no. of reflections (ellipsoidal)*^a^*	298,683 (16,041)
No. of unique reflections (ellipsoidal)*^a^*	24,308 (1215)
Average multiplicity*^a^*	12.3 (13.2)
Completeness (%) (ellipsoidal)*^a^*	92.9 (61.7)
*I*/σ <*I*> (ellipsoidal)*^a^*	13.0 (1.5)
*R*_meas_	0.121 (1.91)
*R*_pim_	0.047 (0.719)
CC½*^a^*	1 (0.367)
Wilson B factor	83.71

**Refinement statistics**	
*R*_work_	0.259
*R*_free_	0.274
Number of nonhydrogen atoms	4849
Macromolecules	4845
Ligand (zinc)	4
Protein residues	611
RMS	
Bonds	0.002
Angles	0.43
Ramachandran (%)	
Favored	96.8
Allowed	3.2
Outliers	0
Rotamer outliers (%)	1.2
Clashscore	5.5
Average B-factor	86.3
Macromolecules	86.3
Ligand (zinc)	95.6
PDB accession code	5W9A

*^a^* These statistics are for data that were truncated by STARANISO to remove poorly measured reflections affected by anisotropy.

*^b^* The resolution limits are shown for each of the three reciprocal lattice axes (a*, b*, and c*). STARANISO has applied an approximately eliptical cutoff to the reflection data.

**Figure 2.**
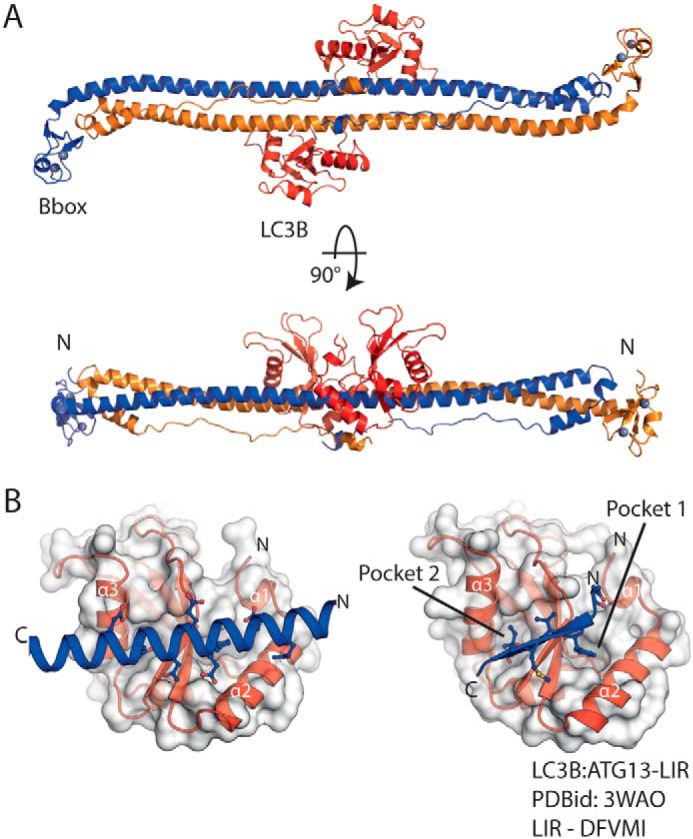
**The coiled coil of Trim5α binds LC3B through a helical motif.** *A*, cartoon representation of the RhT5 88–296 EK/RD–LC3B protein complex. Chain A/B (*blue*/*orange*) form the Trim5α antiparallel coiled coil dimer, LC3B molecules are *red*, and zinc atoms are shown as *spheres* (*silver*). *B*, expanded view of the LC3B-binding site (*left*) and comparison with a typical β-strand LIR motif (LC3B–ATG13-LIR PDB code 3WAO). Both the helical and β-strand LIR occupy and proceed in the same orientation through the LC3B-binding site.

The two RhT5 88–296 EK/RD monomers are arranged as an elongated antiparallel dimer as seen previously (Fig. 2*A*). Comparison of the refined model with our previous structure of the Trim5α Bbox and coiled-coil reveals a high degree of structural similarity with an root-mean-square deviation of 1.1 Å across equivalent Cα atoms (Fig. S1). There is no evidence of flexibility in the coiled-coil between this model and the previously determined structure as was observed when comparing other structures of Trim protein coiled-coils (17, 18).

The two LC3B molecules adopt the typical ubiquitin-like fold and are highly similar to previously determined structures (root-mean-square deviation 0.9 Å to PDB code 2LUE) (Fig. S1). Within the crystal they are positioned toward the center and on either side of the Trim5α coiled-coil. Each LC3B monomer makes essentially identical interactions with a single RhT5 88–296 EK/RD monomer with no interactions between the two LC3B molecules.

At the interface, the bound section of the long Trim5α helix occupies the same groove on the LC3B surface that is used by a typical LIR interaction. Moreover, it is presented in same orientation running parallel with strand β2 of from LC3B (Fig. 2*B*). The Trim5α–LC3B interface buries ∼825 Å^2^ of surface area of each molecule, corresponding to 5% of the RhT5 88–295 EK/RD surface area and 11% of the LC3B surface area. An electrostatic surface calculation (Fig. S2) shows an area of strong negative charge located in the center of the Trim5α coiled coil that is complementary to the general positive charge of the LC3B surface. Residues involved in the interaction span the residue range of Gln^189^–Glu^210^ in Trim5α.

As seen for a typical LIR motif, the interaction centers around Trim5α side-chain interactions in the large hydrophobic pocket of LC3B. Here, it is the side chain of Trim5α Trp^196^ that protrudes away from the coiled coil and occupies the large hydrophobic pocket of LC3B. By contrast, the second hydrophobic pocket that is typically occupied by a small hydrophobic residue is unoccupied in our structure. Instead, the side chain of Gln^203^ is located above the pocket with the side chain amide making a hydrogen bond with the backbone carboxyl of Leu^53^ in strand β2 of LC3B.

**Figure 3.**
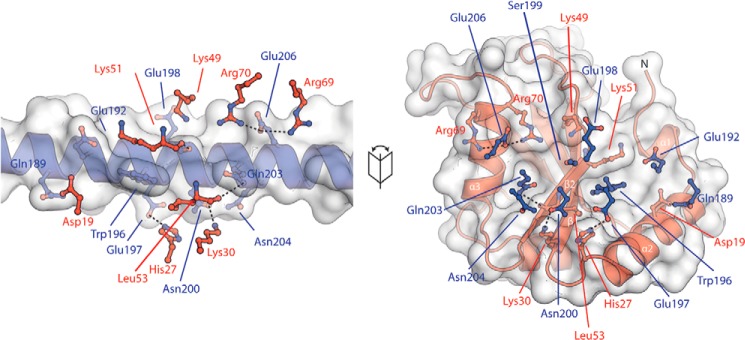
**Interactions mediating binding of LC3B to the Trim5α coiled coil.** A fold out of the LC3B–RhT5α interaction with RhT5α in *blue* and LC3B in *red* is shown. Residues from the opposing molecule are shown as ball-and-stick representation with potential hydrogen bonds as *dashed lines*. Trp^196^ of Trim5α occupies pocket 1 of LC3B.

In addition, there are further interactions outside the hydrophobic pockets that are mediated by acidic residues on the Trim5α coiled-coil. Glu^192^ forms a salt bridge with Lys^51^ on strand β2 of LC3B, Glu^197^ forms a hydrogen bond with His^27^ at the C terminus of helix α2 from LC3B, and Glu^206^ forms potential salt bridges with Arg^69^ and Arg^70^ at the C terminus of helix α3 (Fig. 3). Further interactions are mediated by Ser^199^ that makes a hydrogen bond with the backbone carboxyl of Lys^51^ and the side-chain amine of Gln^189^ that makes a hydrogen bond with the side chain of Asp^19^ on helix α2 of LC3B.

### Mutational analysis of the Trim5α–LC3B interface

**Figure 4.**
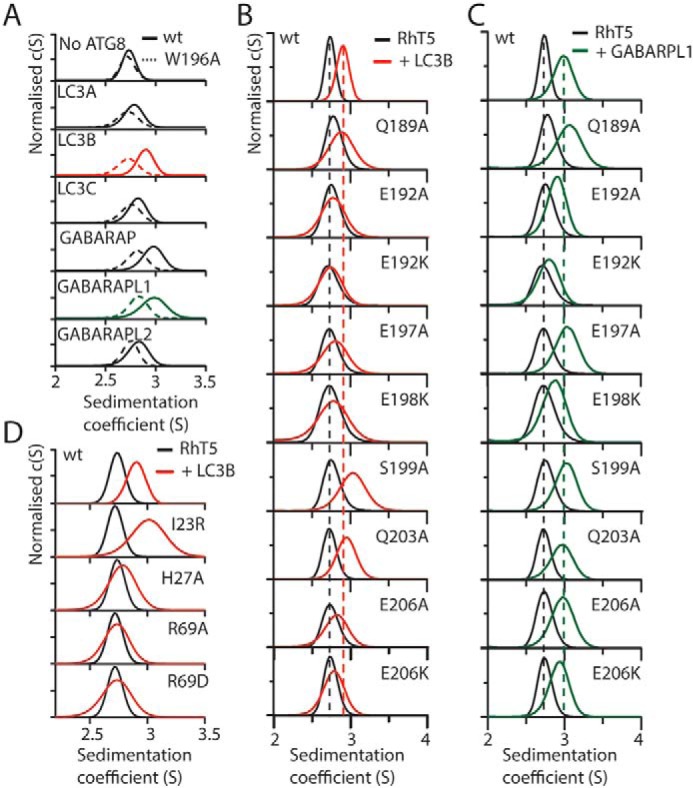
**Mutational analysis of the LC3B-RhT5α interface.** *A*, *c*(*s*) analysis of the RhT5 88–296 EK/RD-mATG8 interaction with all mATG8 proteins at 20 μm equimolar concentration, WT (*solid lines*), and W196A RhT5 88–296 EK/RD (*dashed lines*). *B* and *C*, *c*(*s*) analysis of mixtures of WT and mutant RhT5 88–296 EK/RD with either LC3B (*B*) or GABARAPL1 (*C*). *Dashed lines* indicate the position of RhT5 88–296 EK/RD (*black*) or with the addition of either LC3B (*red*) or GABARAPL1 (*green*). *D*, *c*(*s*) analysis of LC3B mutants (*red*) mixed with WT RhT5 88–296 EK/RD (*black*).

To investigate the importance of specific residues in the Trim5α–LC3B interface, we undertook site-directed mutagenesis to probe the contribution of key residues in the interface and assessed complex formation by SV–AUC. In all assays LC3B and mutants of RhT5 88–296 EK/RD were mixed at an equal concentration of 20 μm. The effects of mutations were judged based upon the magnitude of the perturbation to the fast moving peak corresponding to the Trim–LC3B complex in the *c*(*s*) analysis. Control size-exclusion chromatography coupled to multiangle laser light scattering (SEC–MALLS) and *c*(*s*) analysis of all Trim5α mutants alone demonstrate that they retain the dimeric assembly and do not show large perturbations of the overall structure of the protein (Fig. S3 and Fig. 4, *B* and *C*, *black lines*).

The Trp^196^ side chain is at the center of the interface filling the large hydrophobic pocket of LC3B. The effect of mutating this residue to alanine was assessed for binding to each of the six mATG8 isoforms. Analysis by the *c*(*s*) distribution showed that mutation of Trp^196^ to alanine abolished binding between the LC3A, LC3B, LC3C, and GABARAPL2, whereas GABARAP and GABARAPL1 still bound but with a reduced affinity (Fig. 4*A*). This suggests that although Trp^196^ was important for the affinity of binding, other residues in the interface are able to maintain the interaction in its absence. To test this hypothesis we probed the role of other Trim5α residues in the interface by mutating them to either an alanine or lysine. Each mutant was assayed against LC3B and GABARAPL1 because these two mATG8s showed the greatest affinity for WT Trim5α.

We first tested residues likely to contribute to the acidic surface charge at the center of the Trim5α coiled coil. Mutations of both Glu^192^ and Glu^206^, that make charge-charge interactions in the interface, to either alanine or lysine disrupt binding to LC3B with the lysine mutation showing the anticipated stronger effect (Fig. 4, *B* and *C*). In GABARAPL1 the alanine mutations have little effect, whereas the E192K lysine disrupts binding the E206K mutation has minimal effect. Mutation of Glu^197^ to alanine, removing the interaction with Gln^26^/His^27^, reduced the binding of LC3B but did not abolish it completely. However, the mutation had no effect on the binding of GABARAPL1. A charge swapping mutation of E198K creating repulsion against Lys^51^ reduced binding against LC3B and GABARAPL1. These mutations demonstrate that the general electrostatic interaction contributes to binding.

In addition to the ionic interactions, three hydrogen bond contacts that contribute to the interface were also identified. Therefore, these interactions were also probed by mutating the residue on Trim5α to alanine. Mutation of Gln^189^, which forms a hydrogen bond to Asp^19^ in LC3B, had a minimal effect on LC3B binding. However, this mutation resulted in an increase in the *S* value of the fast moving peak for GABARAPL1 binding, suggesting a stronger interaction. Structure comparison of GABARAPL1 with LC3B shows a glutamate at position 19 (compared with the aspartate in LC3B), which would likely clash with Gln^189^. Therefore, the observed increase in GABARAPL1 binding may result from the alanine substitution relieving this clash.

In a similar manner, mutation of Gln^203^ to alanine was found to increase the affinity of binding to LC3B but did not affect binding to GABARAPL1. Gln^203^ is located at the edge of the small hydrophobic pocket and forms a hydrogen bond with the backbone of Leu^53^. Mutation to alanine would remove this hydrogen bond and was predicted to weaken and not strengthen the interaction. However, it may be that the loss of a hydrogen bond is compensated for by accommodation of the alanine residue in the previously unoccupied L pocket on LC3B.

In addition to interface residues in the Trim5α coiled coil, we identified three residues on LC3B that were probed by mutagenesis for their contribution to the binding of Trim5α. Mutation of LC3B His^27^ to alanine removes a hydrogen bond formed with Glu^197^ on Trim5α and has the similar effect to the RhT5 E197A mutant (Fig. 4*D*). Substitution of LC3B Arg^69^ with either alanine or aspartate is predicted to disrupt ionic interactions with Glu^210^ and Glu^206^ of Trim5α and abolished binding. In the large hydrophobic pocket, Ile^23^ is packed adjacent to Trp^196^ of Trim5α. Mutation to an arginine was predicted to occlude binding into this pocket by filling the same space as occupied by the tryptophan side chain. Surprisingly, this mutation resulted in a faster moving peak in the SV–AUC analysis, suggesting a stronger interaction. However, given the guanidinium head group of arginine residues can pack in a π-stacking conformation with aromatic side chains, this mode of interaction with Trp^196^ provides a possible explanation for the observed increase in binding.

Previous studies have demonstrated an interaction between Trim5α and components of the autophagy machinery, including members of the ATG family. Furthermore microscopy experiments have demonstrated co-localization of Trim5α in autophagic structures in cells (31). The role of autophagy in restriction is less clear with conflicting results reported (9, 12, 31). To assess whether the interaction with the mATG8s play a key role in the restriction of retroviral infection, we undertook restriction assays to examine the effect of W196A and E197A mutations on restriction by either Trim5α or TrimCyp. The inclusion of either mutation individually or the double mutant had no effect on the infectivity of HIV-1 in our restriction assay (Table 2). Both Trim5α and TrimCyp exhibit a secondary block to infection in the presence of the proteasome inhibitor MG132. This block occurs after the completion of reverse transcription but prior to integration of the provirus. The inclusion of proteasome inhibitor at either 1 or 16 μg/ml had no effect on the infectivity of virus in our assay. These results are in agreement with those published by Imam *et al.* (31). This suggests that although Trim5α is able to bind LC3B and members of the mammalian ATG8 family, they do not contribute to the restriction of retroviral infection at either the primary block prior to reverse transcription or the secondary block after reverse transcription.

**Table 2:** **Restriction assay of Trim5 and TrimCyp mutants in the presence and absence of proteasome inhibitor MG132** The cells were transduced with vectors expressing YFP and either TRIM5, TRIMCyp, or their mutants before challenging with HIV-1 expressing GFP, in the presence or absence of MG132. The cells were analyzed by flow cytometry 48 h after challenge. The numbers are ratios of percentages of infected cells containing restriction factor to percentages of infected cells that do not contain restriction factor.

	Without MG132	1 μg/ml MG132	16 μg/ml MG132
Trim 5α	0.12 ± 0.06	0.14 ± 0.04	0.12 ± 0.01
Trim 5α W196A/E197A	0.10 ± 0.05	0.11 ± 0.05	0.09 ± 0.03
Trim 5α E197A	0.12 ± 0.03	0.12 ± 0.04	0.19 ± 0.03
TrimCypA	0.10 ± 0.06	0.10 ± 0.02	0.09 ± 0.02
TrimCypA W196A	0.08 ± 0.03	0.08 ± 0.05	0.05 ± 0.03
TrimCypA W196A/E197A	0.10 ± 0.06	0.11 ± 0.07	0.05 ± 0.01
TrimCypA E197A	0.12 ± 0.05	0.13 ± 0.08	0.11 ± 0.03

## Discussion

During autophagy members of the mATG8 family play a crucial role in phagophore formation and expansion. Modification of LC3B, and other mATG8s, via cleavage of the C terminus and the subsequent conjugation of a phosphatidylethanolamine lipid to the C terminus, is a key marker of autophagosome formation. This modification anchors the mATG8s to the autophagosome where they act as an adaptor, tethering substrate proteins, including components of the autophagosome maturation pathway and selective autophagy receptors, to the autophagosomal membrane.

Previously characterized interactions between ATG8 proteins and their binding partners show a conserved mode of interaction (3) comprising a linear binding motif with the consensus sequence of [DEST][WFY]-{RGKP}{RGKP}-[ILV] arranged as a β-strand with the large and small hydrophobic residues located in pockets on the surface of the ATG8 protein. The structure now presented here demonstrates a second mode of binding where a helical motif occupies the same binding groove on the surface of LC3B with the large hydrophobic pocket similarly occupied.

Based upon our structural observations and biophysical characterization of this interaction we propose a “helical LIR” motif, where upon accounting for the difference in residue spacing imposed by the helical secondary structure an equivalent consensus sequence is accommodated. This new motif would have a consensus sequence of an acidic residue with a three-amino acid spacer N-terminal to the large aromatic amino acid and then a further six amino acids N-terminal to the residue that occupies the small hydrophobic pocket ([DEST]-*X*_3_-[WFY]-*X*_6_-[LIVQ]). This results in key residues being presented along a single face of an α-helix. In the case of Trim5α the residues Glu^192^, Trp^196^, and Gln^203^ fulfill these positions, with Gln^203^ located on the edge of the small hydrophobic pocket. Mutation of these residues alters binding, either decreasing the affinity or in the case of Gln^203^ substitution with a small hydrophobic residues results in an increase in binding. Although these residues are located in key positions in the binding groove, our data demonstrate that other residues present on the helix contribute to binding. This is consistent with the increased surface area and number of residues presented by the helix, 10 residues burying ∼820 Å^2^ compared with 5 amino acids burying ∼610 Å^2^ for a typical LIR motif. Furthermore, mutation of residues in the helical LIR contribute differently to binding of LC3B and GABARAPL1, suggesting subtle differences in the recognition of different ATG8 proteins.

To examine further features that contribute to the LC3B-binding site, we aligned the coiled-coil region of Trim5α from 57 species with unique sequences present in the UniProt database (Fig. S4). It is evident that although there are conserved residues that we have shown contributing to binding, other amino acids are less well-conserved. Of note, Trp^196^ is not strictly conserved across the Trim5α of all species. Mapping sequences to an evolutionary tree, we observe a clear sequence division at this position between the new- and old-world monkeys. This divergence maps to after the separation between the new- and old-world monkeys that occurred during the Oligocene era, between 30 and 40 million years ago and prior to the divergence of the apes and old-world monkeys ∼10–20 million years ago (32), suggesting that a tryptophan at this position has been acquired and retained.

**Figure 5.**
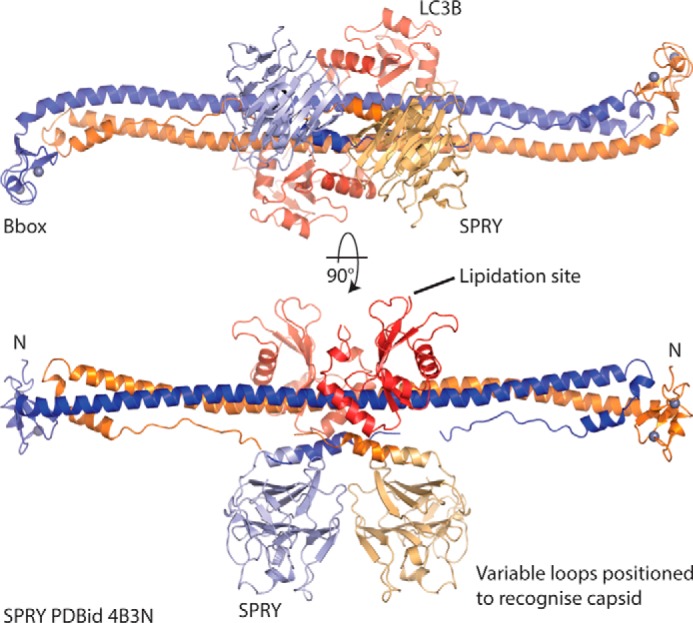
**Model of Trim5α–LC3B complex.** The SPRY domains from Rhesus Trim5α (PDB code 4B3N) are positioned on the Trim5α Bbox coiled coil (PDB code 4TN3) by superposition of common residues. The structure of the RhT5α–LC3B complex (this work) was then superimposed on 4TN3 to position the LC3B molecules. This model positions the variable loops of the SPRY domain to recognize capsid, whereas the lipidation site at the C terminus of LC3B is available to be incorporated in the autophagosomal membrane.

Based upon previously determined structures of the Trim5α PRY/SPRY domain (33, 34) and an overlap with residues in the L2 linker region that are present in structures of both the PRY/SPRY domain (34) and the Bbox coiled-coil region of Trim5α (16), it is possible to construct a model of the Trim5α molecule that positions the SPRY domain from each monomer adjacent to one another at the center of the coiled-coil region (16, 35–37). This model positions the variable loops of the SPRY domain to recognize the retroviral capsid, and the Bbox and RING domains are available for higher-order assembly and ubiquitylation. Inclusion of LC3B into this model using our current structure (Fig. 5) now positions the LC3B either side of the center of the coiled-coil, adjacent to the SPRY domains, without interfering with the positioning of the SPRY domains or making interactions with regions of the L2. Further examination of the orientation of the LC3B relative to the SPRY domain positions the C termini of the LC3Bs on the opposite side of the coiled coil. This demonstrates that LC3B could be accommodated within the Trim5α higher-order assembly and would allow the SPRY domain to remain accessible to recognize substrates, whereas lipidated LC3B could tether Trim5α to the autophagosomal membrane.

Comparison of the affinity of interaction of the helical LIR with that of the typical LIR–LC3B interactions suggests that the LC3B-Trim5α interaction is at the lower end of reported affinities, with typical interaction in the *K_D_* range of 1–50 μm range (38). However, because both LC3B molecules present in our model are oriented with the C terminus exposed, we would expect a single Trim5α dimer to bind two LC3B molecules. With LC3B tethered on the autophagosomal membrane, this would generate an avid interaction and greatly amplify the strength of binding.

Members of the TRIM protein family have an emerging role in autophagy. The structure presented here provides the first molecular insight into the interaction of TRIM proteins with components of the autophagy machinery. This interaction proceeds through a helical motif and alters the current paradigm of binding to members of the ATG8 protein family.

## Experimental procedures

### Protein purification

Mammalian ATG8 and Trim5α constructs were inserted into pET47 and expressed with an N-terminal His tag in *E. coli* LOBSTR BL21(DE3) cells. The proteins were purified by immobilized nickel-affinity chromatography, and the His tag was removed by incubation with HRV 3C protease prior to anion exchange (6 ml of Resource Q) and size-exclusion chromatography using an Superdex 200 (16/60) column equilibrated with 10 mm Tris/HCl, pH 7.8, 150 mm NaCl, 0.1 mm TCEP. Proteins were concentrated to 20 mg/ml using Vivaspin concentrators and stored at −80 °C until required.

### Analytical ultracentrifugation

All sedimentation velocity analytical ultracentrifugation experiments were carried out at 20 °C (293 K) using a Beckman Coulter model XL-I with absorbance optics in double sector charcoal filled Epon center pieces. All samples were centrifuged in a Beckman Coulter eight-hole An-50 Ti rotor at 50,000 rpm. Prior to the experiments all samples were exhaustively dialyzed against a buffer containing 10 mm Tris/HCl, pH 8, 150 mm NaCl, and 0.1 mm TCEP. SEDNTERP (39) was used to determine a solvent density of 1.005 g ml^−1^ and a viscosity of 0.01021 cp. The data were analyzed using a continuous *c*(*s*) distribution in SEDFIT (39). Because many experiments contained mixtures of two proteins, each with a unique partial specific volume, a constant value of 0.73 ml g^−1^ was used for all samples.

### Crystallization and structure determination

The Trim5α construct RhT5 88–296 E120K/R121D and LC3B construct 2–119 were mixed at equimolar concentrations (330 μm) and crystallized by the vapor diffusion method at 290 K. The protein mixture was combined with a precipitant-mixture containing 0.2 m NH_4_Cl, 0.1 m Tris/HCl, pH 8, 20% PEG 6,000 at a ratio of 1:1 and allowed to equilibrate. Large plate crystals formed over a period of ∼48 h.

The crystals were harvested into a cryoprotectant containing the precipitant mixture supplemented with 20% glycerol and flash-frozen in liquid nitrogen. Diffraction experiments were conducted at the Australian synchrotron. The data were collected at 9900 eV.

The data were indexed with XDS (40), and initial merging and scaling with AIMLESS (41) indicated significant anisotropy in the strength of diffraction. Thus an anisotropic resolution cutoff was applied to the data by the STARANISO server (Global Phasing Limited) giving a maximum resolution of 2.74 Å. The structure was then determined by molecular replacement in PHASER (42) using the Trim5α dimer (PDB code 4TN3) and a single LC3B molecule (PDB code 3WAO) as search models. The second LC3B molecule was generated by noncrystallographic symmetry, rotating the model 180° and aligning based on the Trim5α dimer. The model was then completed using iterative rounds of manual model building in COOT (43) and refinement in PHENIX (44). The data collection and refinement statistics are presented in Table 1.

### SEC–MALLS

SEC–MALLS was used to determine the molecular weight and oligomeric state of RhT5 88–296 EK/RD and LC3B mutants. Samples (100 μl) were applied to a Superdex 75 Increase 10/300 column in a running buffer containing 10 mm Tris/HCl, pH 7.8, 150 mm NaCl, 0.1 mm TCEP, and 3 mm azide. The MALLS unit comprised a Dionex HPLC with a PSS SLD7000 7-angle MALLS detector and a Shodex RI-101 differential refractive index detector. The data were analyzed using the PSS winGPC Unichrom software package.

### Restriction assays

CRFK cells were transduced with vectors expressing YFP and either TRIM5, TRIMCyp, or their mutants before challenging with HIV-1 expressing GFP, in the presence or absence of either 1 μg/ml MG132 or 16 μg/ml MG132. The cells were analyzed by flow cytometry 48 h after challenge. The numbers are ratios of percentages of infected cells containing restriction factor to percentages of infected cells that do not contain restriction factor. A ratio of less than 0.3 was taken to indicate restriction (45).

## Author contributions

J. R. K. and D. C. G. conceptualization; J. R. K. and D. C. G. formal analysis; J. R. K., M. M. B., A. F., M. W. Y., M. J. B., F. G. P., and J. P. S. investigation; J. R. K., M. W. Y., J. P. S., and D. C. G. writing-original draft; J. R. K. and D. C. G. writing-review and editing; F. G. P. resources; J. P. S. supervision.

## Supplementary Material

Supporting Information

## Footnotes

3 The abbreviations used are:

mATG8: mammalian ATG8
LIR: LC3-interacting region
SV–AUC: sedimentation velocity analytical ultracentrifugation
PDB: Protein Data Bank
SEC–MALLS: size-exclusion chromatography coupled to multiangle laser light scattering
TCEP: tris-(2-carboxyethyl) phosphine hydrochloride.
