# Supplementary material for: A helical LC3-interacting region mediates the interaction between the retroviral restriction factor Trim5α and mammalian autophagy-related ATG8 proteins
Source: J Biol Chem. 2018 Oct 3;293(47):18378–86. doi: 10.1074/jbc.RA118.004202 (PMC6254359; doi:10.1074/jbc.RA118.004202)
Supplement: Supporting Information [file supp_293_47_18378__index.html]

A helical LIR mediates the interaction between the retroviral restriction factor Trim5α and the mammalian autophagy related ATG8 proteins — Trim5α binds to LC3B via a helical LIR motif — A helical LC3-interacting region mediates the interaction between the retroviral restriction factor Trim5α and mammalian autophagy-related ATG8 proteins — Trim5α binds to LC3B via a helical LIR motif — Supporting Information 

# A helical LC3-interacting region mediates the interaction between the retroviral restriction factor Trim5α and mammalian autophagy-related ATG8 proteins

## Supporting Information

- Supporting Information (to be published online) - Supplementary figures S1-S4
