# Supplementary material for: A helical LC3-interacting region mediates the interaction between the retroviral restriction factor Trim5α and mammalian autophagy-related ATG8 proteins
Source: J Biol Chem. 2018 Oct 3;293(47):18378–86. doi: 10.1074/jbc.RA118.004202 (PMC6254359; doi:10.1074/jbc.RA118.004202)
Supplement: Supporting Information [file supp_RA118.004202_138454_1_supp_200100_pzv2y4.pdf]

A helical LIR mediates the interaction between the retroviral restriction factor Trim5α and the mammalian autophagy relation ATG8 proteins

Jeremy R. Keown[1,#], Moyra M. Black[1], Aaron Ferron[2], Melvyn Yap[2], Michael J. Barnett[1], F. Grant Pearce[3], Jonathan P. Stoye[2], David C. Goldstone[1,4]*

From the [1]School of Biological Sciences, University of Auckland, Auckland, New Zealand [2]The Francis Crick Institute, London, UK [3]School of Biological Sciences, University of Canterbury, Christchurch, New Zealand. [4]Associate Investigator, Maurice Wilkins Centre for Molecular Biodiscovery

Running title: *Trim5α binds to LC3B via a helical LIR motif*

[#]Present address: Division of Structural Biology, University of Oxford, Oxford, UK

[*]To whom correspondence should be addressed: David C. Goldstone: School of Biological Sciences, University of Auckland, Auckland, New Zealand; d.goldstone@auckland.ac.nz; Tel (+649) 923-4607.

***Keywords**:* Autophagy, Trim5α, TRIM protein family, LC3B, LIR, tripartite motif-containing, retroviral restriction factor, autophagosome, antiviral protein.

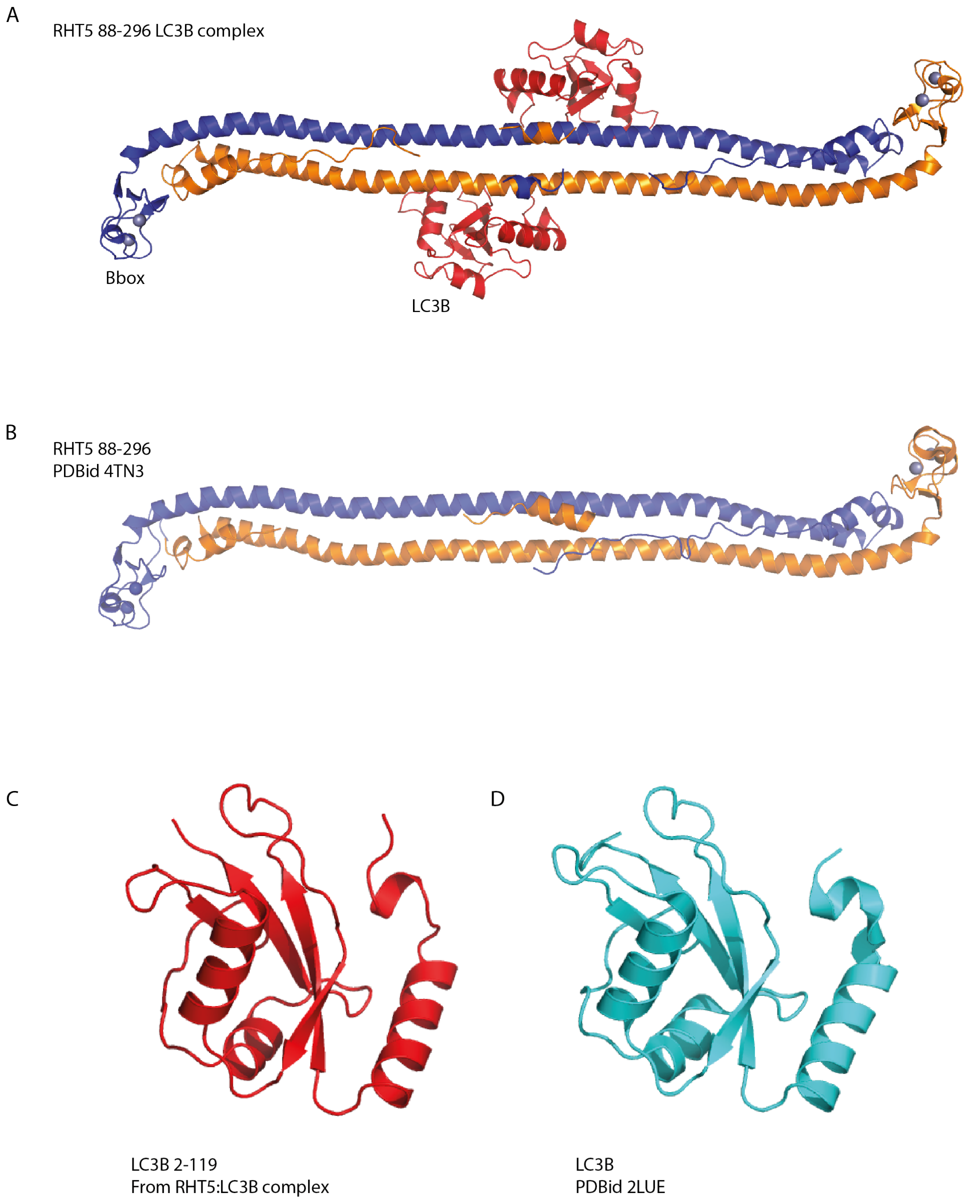

Supplementary Figure S1

Cartoon representation of the Trim5α coiled-coil are presented, this manuscript (A) and that previously determined (PDBid 4TN3) (B) showing them to be highly similar. Comparison of the LC3B structure determined in this work (C) with previously determined structure of LC3B (PDBid:2LUE) (D)

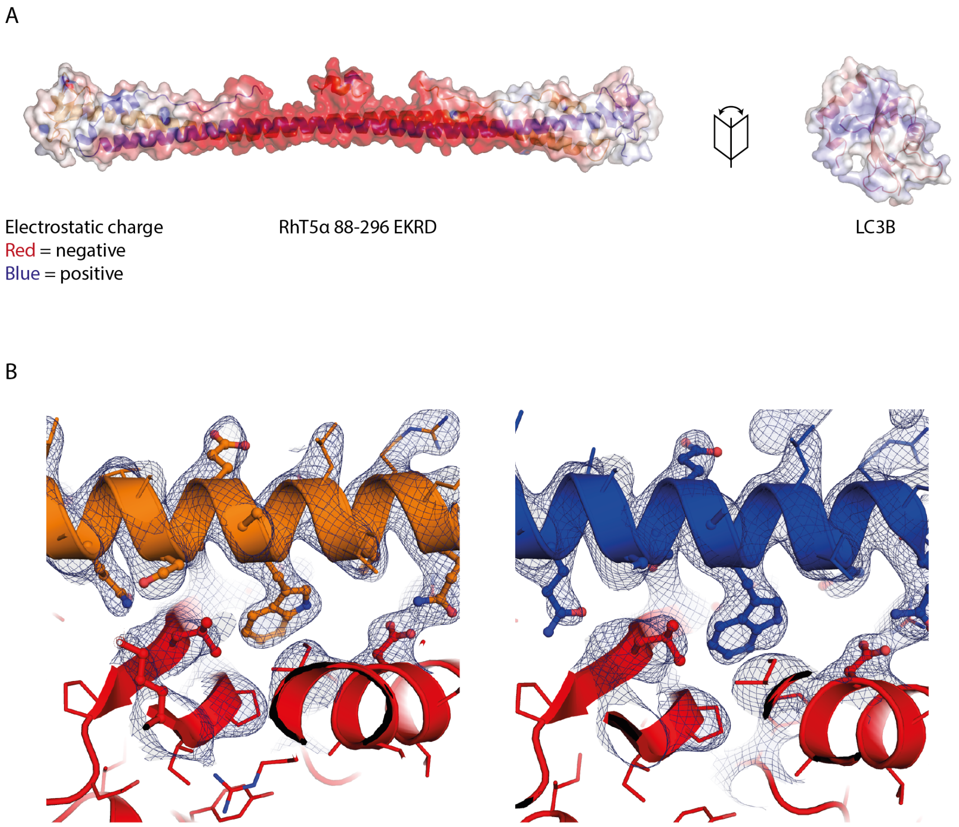

Fig S2

Supplementary Figure S2

A) Electrostatic surface analysis of Trim5α and LC3B calculated using APBS within the PyMOL interface. The electrostatic surface is coloured from –10 to +10. B) Representative electron density for the Trim5α-LC3B binding site. Density is 2fo-fc contoured at 1σ. Each panel shows the density for one interaction found in the Asymmetric unit.

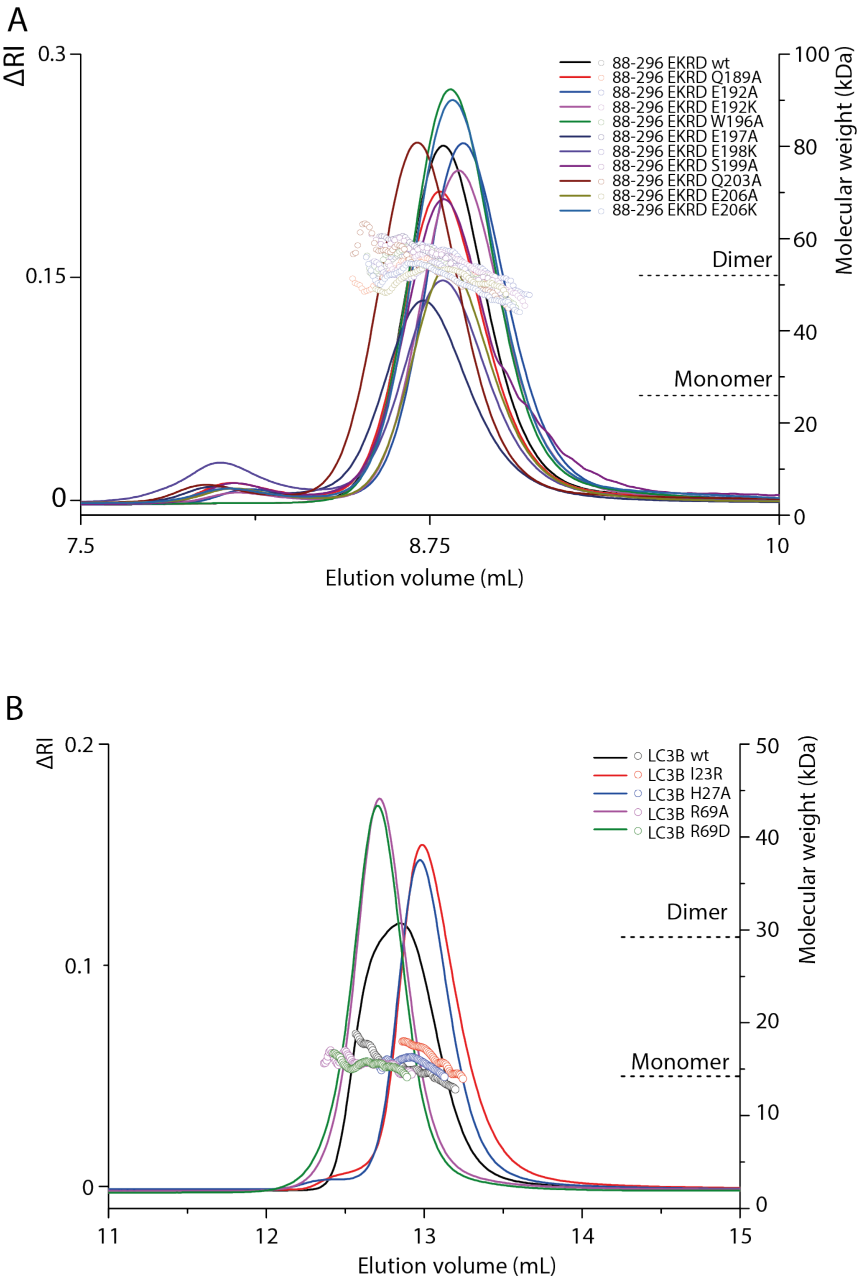

Supplementary Figure S3

Size exclusion chromatography coupled to multi angle laser light scattering (SEC-MALLS) of RhT5 88-296 EK/RD (A) and LC3B (B) for wildtype and mutant proteins showing their oligomeric state is unaffected by the mutations. Solid lines show the differential refractive index signal, circles are calculated weight averaged molecular weight.

A

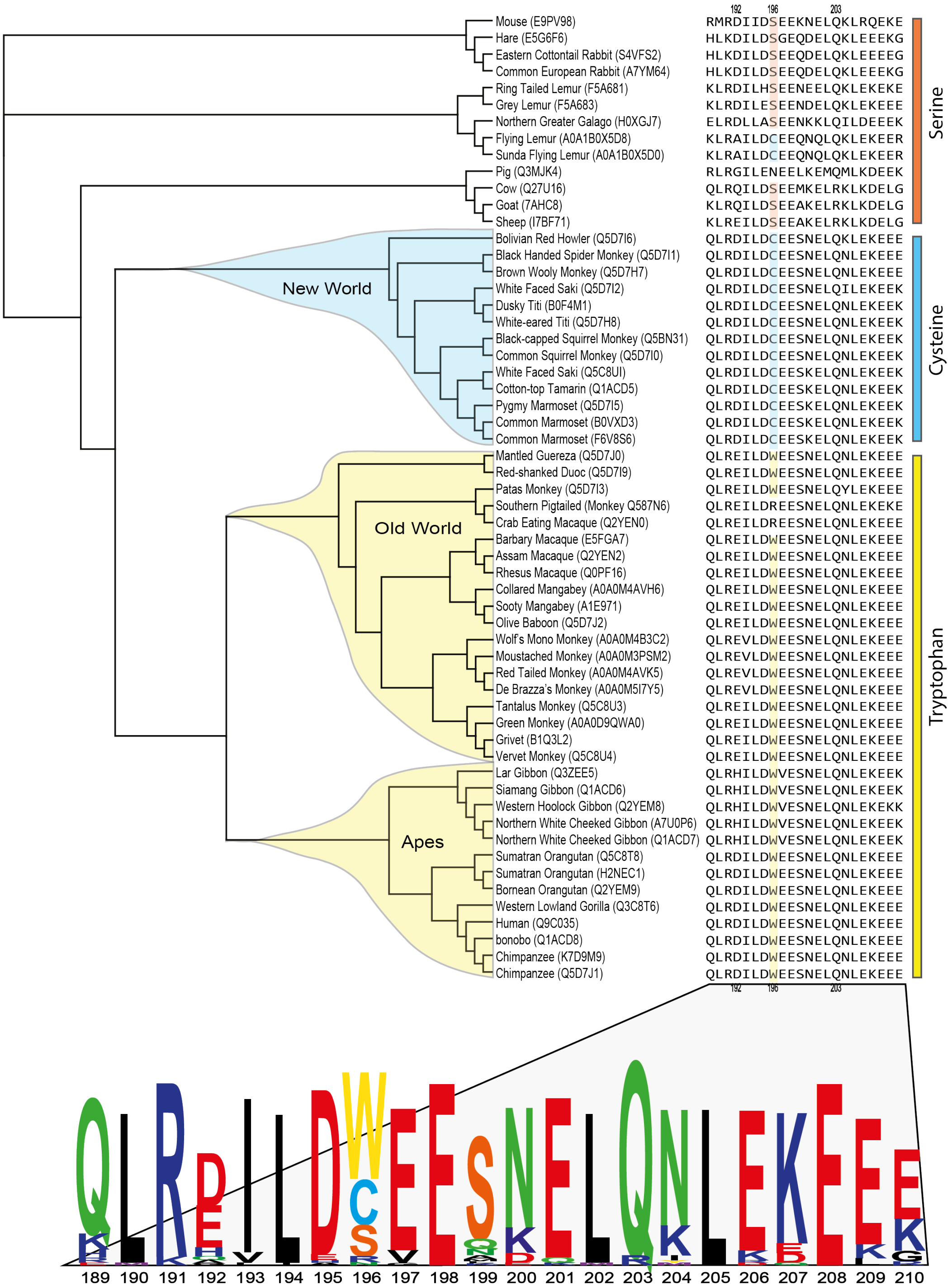

Supplementary Figure S4

Bioinformatic analysis of Trim5α proteins, sourced from the uniprot database. A phylogenetic tree generated from 58 Trim5α species partitions Trim5α sequences based pon evolutionary groups. Elements of the helical LIR motif partition with the divergence of species on an evolutionary timescale with the acquisition and retention of a tryptophan residue at position 196. A HMM LOGO for the helical LIR region is shown demonstrating the relative conservation of each position.
